# Effect of gender, age and vaccine on reactogenicity and incapacity to work after COVID-19 vaccination: a survey among health care workers

**DOI:** 10.1186/s12879-022-07284-8

**Published:** 2022-03-26

**Authors:** Irit Nachtigall, Marzia Bonsignore, Sven Hohenstein, Andreas Bollmann, Rosita Günther, Cathrin Kodde, Martin Englisch, Parviz Ahmad-Nejad, Alexander Schröder, Corinna Glenz, Ralf Kuhlen, Petra Thürmann, Andreas Meier-Hellmann

**Affiliations:** 1 Infectious Diseases, HELIOS Hospital Emil-Von-Behring, Walterhöferstr. 11, 14165 Berlin, Germany; 2 Institute of Hygiene and Environmental Medicine, Charité-Universitätsmedizin Berlin, Hindenburgdamm 27, 12203 Berlin, Germany; 3 Center for Hygiene, Evangelische Kliniken Gelsenkirchen, Munckelstraße 27, 45879 Gelsenkirchen, Germany; 4 Heart Center Leipzig at University of Leipzig and Leipzig Heart Institute, Russenstraße 69a, 04289 Leipzig, Germany; 5 Helios Kliniken GmbH, Friedrichstr. 136, 10117 Berlin, Germany; 6 Department of Pneumology, Lungenklinik Heckeshorn, Helios Klinikum Emil von Behring, Walterhöferstr. 11, 14165 Berlin, Germany; 7 Institute for Medical Laboratory Diagnostics, Center for Clinical and Translational Research, Helios University Hospital Wuppertal, University of Witten/Herdecke, Heusnerstraße 40, 42283 Wuppertal, Germany; 8 Helios Health GmbH, Friedrichstr. 136, 10117 Berlin, Germany; 9 Philipp Klee-Institute for Clinical Pharmacology, Helios University Hospital Wuppertal, Heusnerstraße 40, 42283 Wuppertal, Germany; 10 Department of Clinical Pharmacology, University Witten Herdecke Faculty of Health Witten, Alfred-Herrhausen-Straße 50, 58455 Witten, Germany

**Keywords:** Vaccination, COVID-19, Sex differences, Circadian rhythm, Reactogenicity, Working capacity

## Abstract

**Background:**

The aim of our study was to assess the impact the impact of gender and age on reactogenicity to three COVID-19 vaccine products: Biontech/Pfizer (BNT162b2), Moderna (mRNA-1273) and AstraZeneca (ChAdOx). Additional analyses focused on the reduction in working capacity after vaccination and the influence of the time of day when vaccines were administered.

**Methods:**

We conducted a survey on COVID-19 vaccinations and eventual reactions among 73,000 employees of 89 hospitals of the Helios Group. On May 19th, 2021 all employees received an email, inviting all employees who received at least 1 dose of a COVID-19 to participate using an attached link. Additionally, the invitation was posted in the group’s intranet page. Participation was voluntary and non-traceable. The survey was closed on June 21st, 2021.

**Results:**

8375 participants reported on 16,727 vaccinations. Reactogenicity was reported after 74.6% of COVID-19 vaccinations. After 23.0% vaccinations the capacity to work was affected. ChAdOx induced impairing reactogenicity mainly after the prime vaccination (70.5%), while mRNA-1273 led to more pronounced reactions after the second dose (71.6%). Heterologous prime-booster vaccinations with ChAdOx followed by either mRNA-1273 or BNT162b2 were associated with the highest risk for impairment (81.4%). Multivariable analyses identified the factors older age, male gender and vaccine BNT162b as independently associated with lower odds ratio for both, impairing reactogenicity and incapacity to work. In the comparison of vaccine schedules, the heterologous combination ChAdOx + BNT162b or mRNA-1273 was associated with the highest and the homologue prime-booster vaccination with BNT162b with the lowest odds ratios. The time of vaccination had no significant influence.

**Conclusions:**

Around 75% of the COVID-19 vaccinations led to reactogenicity and nearly 25% of them led to one or more days of work loss. Major risk factors were female gender, younger age and the administration of a vaccine other than BNT162b2. When vaccinating a large part of a workforce against COVID-19, especially in professions with a higher proportion of young and women such as health care, employers and employees must be prepared for a noticeable amount of absenteeism. Assuming vaccine effectiveness to be equivalent across the vaccine combinations, to minimize reactogenicity, employees at risk should receive a homologous prime-booster immunisation with BNT162b2.

*Trial registration:* The study was approved by the Ethic Committee of the Aerztekammer Berlin on May 27th, 2021 (Eth-37/21) and registered in the German Clinical Trials Register (DRKS 00025745). The study was supported by the Helios research grant HCRI-ID 2021-0272.

## Background

Since the WHO declared COVID-19 a pandemic in January 2020, the development and application of vaccines against the virus was classified as an important cornerstone in the fight against the virus. On December 21st, 2020 Biontech and Pfizer received authorization in the European Union for their mRNA COVID-19 vaccine (BNT162b2), followed on January 6th, 2021 by Moderna (mRNA vaccine mRNA-1273) and on January 29th by AstraZeneca (vector vaccine ChAdOx).

After reports of thrombotic events following ChAdOx vaccinations [1, 2], several European countries restricted the vector vaccine to persons above a defined age limit [3]. For younger persons who already received a prime immunisation with ChAdOx, a heterologous boost immunisation with an mRNA vaccine (BNT162b2 or mRNA-1273) was recommended [4].

Physical complaints after vaccinations are differentiated in reactogenicity and adverse effects. Reactogenicity refers to symptoms caused by an (excessive) inflammatory response to vaccination, and can include redness, swelling or pain at the injection site, as well as systemic symptoms, such as fever, myalgia, or headache. Reactogenicity is common and similar in all vaccines. Adverse events refer to disorders, which could potentially be caused, triggered or worsened at any time after vaccination. Type and frequency of reactogenicity and adverse effects related to the COVID-19 vaccines have been described in the studies performed for regulatory approval [5–7]. However, no information was provided regarding possible diverging results related to sex (i.e., due to genetic and hormonal factors) or gender (i.e., due to behavioural and lifestyle aspects). First real-world data shows a higher occurrence of physical complaints in women [8, 9]. Few studies have addressed the safety of the newly applied heterologous vaccinations so far, showing contradictory results [10–13]. So far, no study has addressed the influence of gender and age on reactogenicity and loss of working days caused by select combinations of COVID-19 vaccines and the influence of the time of day at which the vaccine was administered.

## Methods

We conducted a survey addressing all employees of the Helios Kliniken Group in Germany (n = 73,000). Helios is a privately owned company with 89 hospitals, ranging from small community structures to university hospitals. Consent of the group’s work council was obtained.

On May 19th, around 73,000 employees with a personal business address received an email from the chief of medicine, inviting all employees who had received at least 1 dose of a COVID-19 vaccine to participate to participate in the survey. The e-mail included a link to a survey tool. Additionally, the invitation was posted in the group’s intranet page as ‘Top News’. All occupational areas were included (medical personnel, administration staff and other). The vaccination campaign started in Germany in December 2020, prioritising first the elderly and soon after health care workers. The campaign was a continuous process; by May, most health care workers had already completed the primary series of COVID-19 vaccine. The number of vaccinated employees was not known: due to data protection rules employers were not allowed to obtain information on COVID-19 vaccinations among their staff. The-mail was sent out only once; the survey remained open until June 21st, 2021.

**Fig. 1:**
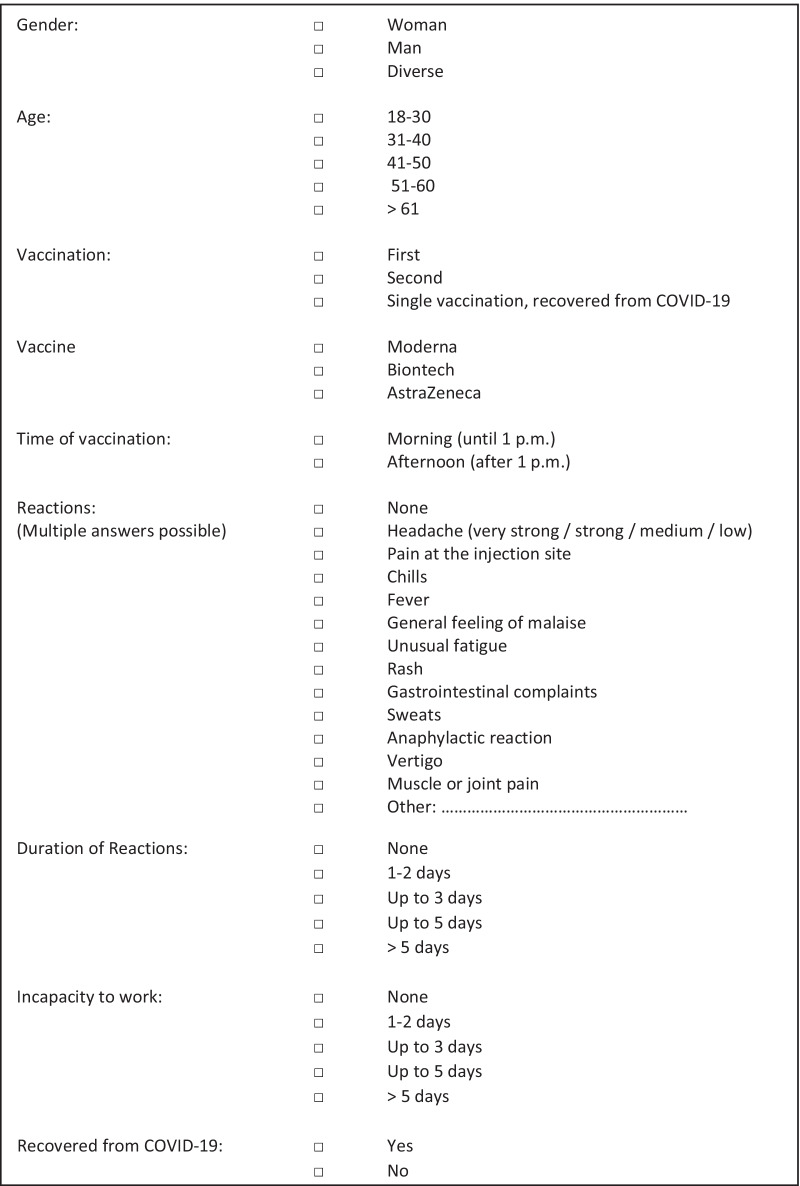
The list of questions in the survey (translated from German)

Participation to the survey was voluntary and non-traceable. A team of infectious diseases experts designed the survey; the translation of the included questions is displayed in Fig. 1. A separate questionnaire was completed following each dose of COVID-19 vaccine that was received. Incapacity to work was assessed using information from the questionnaire only, as was self-reported gender.

We classified physical complaints reported by the participants into two grades:

A. Significant impairment: (at least one symptom)

- Headache very strong/strong,
- chills, fever and general feeling of malaise

B. Mild impairment

- Headache mild/low
- pain at injection site, gastrointestinal complaints, skin rash, unusual fatigue, sweats, vertigo, muscle or joint pain and other.

To clarify that the classification of the impairment into significant and mild is based on our interpretation, the adjective assumed is prefixed in the results.

### Statistics

For the description of the respondent characteristics and outcomes, we employed χ^2^ tests for binary variables. Effects were estimated in the R environment for statistical computing (version 4.0.2, 64-bit build).

For the multivariable analysis of the ordinal scales outcomes (a) grade of impairment (none < mild < significant) and (b) duration of the incapacity for work (none < 1 or 2 days < 3 or more days) we used ordered logistic regression (proportional odds logistic regression). We specified custom contrasts for the predictor variables [14]. All variables entered the models as (one or multiple) contrasts comparing the current factor level to the baseline. The intercept represented the grand mean.

For the multivariable analysis of the ordinal scales outcomes (a) frequency of significant impairment (none < significant impairment after either vaccination < significant impairment after both vaccinations) and (b) frequency of incapacity to work (none < incapacity to work of minimum 1 day after either vaccination < incapacity to work of minimum 1 day after both vaccinations) we used ordered logistic regression. With the exception of the combination of vaccines, the specification of contrasts was identical to the others models. The combination of vaccines entered the models as repeated contrasts (sliding differences) comparing adjacent levels (the current level to the preceding one). For all tests we applied a two-tailed 5% error criterion for significance.

## Results

In total, 8375 of approximately 73,000 employees of the Helios Group participated in the survey and provided for information on 16,727 vaccinations. 80 participants did not provide any information on their gender, 26 declared to be diverse. Due to the low amount of diverse participants, no separate analysis was carried out for this subgroup. Of the remaining 8269 participants, 74.1% (6131/8269) were women; the proportion of women among all Helios employees by the time of the survey was 76.1%. The characteristics of the participants are given in Table 1. The most common prime-boost vaccination was the homologous schedule with BNT162b2 (50.6%, 4179/8246), followed by the heterologous schedule ChAdOx + BNT162b2 (17.7%, 1465%8246).

**Table 1:** Characteristics of participants

	Women	Men	P-value	Odds ratio (95% confidence interval)
Participants (total = 8375)	74.1% (6131/8269)	25.9% (2138/8269)	< .01	
Age				
18–30	14.0% (859)	11.1% (237)	< .01	
31–40	21.4% (1314)	23.5% (503)	.05	
41–50	24.4% (1495)	25.7% (549)	ns	
51–60	31.1% (1908)	28.4% (607)	.02	
> 61	7.2% (440)	8.7% (187)	.02	
Missing or invalid data	1.9% (115)	2.6% (55)	ns	
Recovered after COVID-19	1.8% (110)	1.4% (28)	ns	
1st vaccine (total = 8269)				
BNT162b2	59.1% (3623)	64.3% (1375)	< .01	
ChAdOx 2	34.4% (2108)	30.0% (641)	< .01	
mRNA-1273	3.3% (203)	2.9% (63)	ns	
Missing or invalid data	3.2% (197)	2.8% (59)	ns	
2nd vaccine (total = 8246)				
BNT162b2a	69.2% (4,244)	69.2% (1,479)	ns	
ChAdOx	9.2% (564)	9.3% (198)	ns	
mRNA-1273	6.1% (371)	6.0% (129)	ns	
Missing or invalid data	15.2% (931)	15.4% (330)	ns	
No 2nd vaccination	0.3% (21)	0.1% (2)	ns	
Vaccine combination, if > 1 vaccination (total = 8246)				
BNT162b2 + BNT162b2	49.5% (3024)	54.1% (1155)	< .01	
mRNA-1273 + mRNA-1273	2.6% (158)	2.3% (49)	ns	
ChAdOx + ChAdOx	9.1% (554)	9.1% (194)	ns	
ChAdOx + BNT162b2	19.0% (1161)	14.2% (304)	< .01	
ChAdOx + mRNA-1273	3.4% (206)	3.7% (78)	ns	
Missing information on 1st or 2nd vaccine	16.5% (1007)	16.7% (356)	ns	
Time of day of vaccination Based on 13,876 vaccinations with information on time of day and gender				
Morning	68.9% (7148)	68.3% (2389)	ns	
Afternoon	31.1% (3230)	31.7% (1109)	ns	
Reactions (multiple answers were possible) Based on 16,207 vaccinations with information on reactions and gender				
None	23.1% (2765)	32.2% (1358)	< .01	1.59 (1.47–1.72)
Headache (very strong / strong)	17.7% (2122)	10.1% (425)	< .01	0.52 (0.47–0.58)
Pain at the injection site	52.2% (6257)	44.8% (1887)	< .01	0.74 (0.69–0.80)
Chills	21.7% (2606)	16.1% (677)	< .01	0.69 (0.63–0.76)
Fever	17.9% (2143)	13.9% (585)	< .01	0.74 (0.67–0.82)
General feeling of malaise	31.4% (3765)	26.5% (1115)	< .01	0.79 (0.73–0.85)
Unusual fatigue	31.4% (3762)	25.3% (1066)	< .01	0.74 (0.68–0.80)
Rash	2.5% (295)	0.7% (28)	< .01	0.27 (0.18–0.39)
Gastrointestinal complaints	5.6% (670)	2.5% (104)	< .01	0.43 (0.35–0.53)
Sweats	9.7% (1169)	7.5% (315)	< .01	0.75 (0.66–0.85)
Vertigo	10.2% (1220)	5.7% (242)	< .01	0.54 (0.47–0.62)
Muscle or joint pain	28.4% (3412)	22.8% (960)	< .01	0.74 (0.68–0.81)
Other	0.8% (814)	3.7% (155)	< .01	0.52 (0.44–0.63)
Anaphylactic reaction	0.3% (31)	0.2% (8)	ns	0.73 (0.34–1.60)
Incapacity to work Based on 13,795 vaccinations with information on incapacity to work and gender				
None	75.3% (7746)	81.9% (2869)	< .01	
1–2 days	18.7% (1924)	14.3% (501)	< .01	
3 or more days	6.0% (622)	3.8% (133)	< .01	

Nearly 75% of vaccinations led to physical complaints (physical complaints were reported for 12,084 of 16,207 vaccinations = 74.6% of all vaccinations); 41.2% of vaccinations (6677/ 16,207) led to complaints that reported a (assumed) significant impairment. The physical complaint most commonly reported was pain at the injection site (50.2%, 8144/16,207), followed by general feeling of malaise (30.1% 4880/16207). Severe adverse effects were reported after nine vaccinations and included petechiae, thromboses and bleeding. Anaphylactic reactions occurred in 0.24% of all vaccinations.

An incapacity to work of one or more days was reported to have followed 23.0% of all vaccinations (3180/13795), 24.7% (2546/10,292 of vaccinations among women compared to 18.1% (634/3503) of vaccinations among men.

**Fig. 2:**
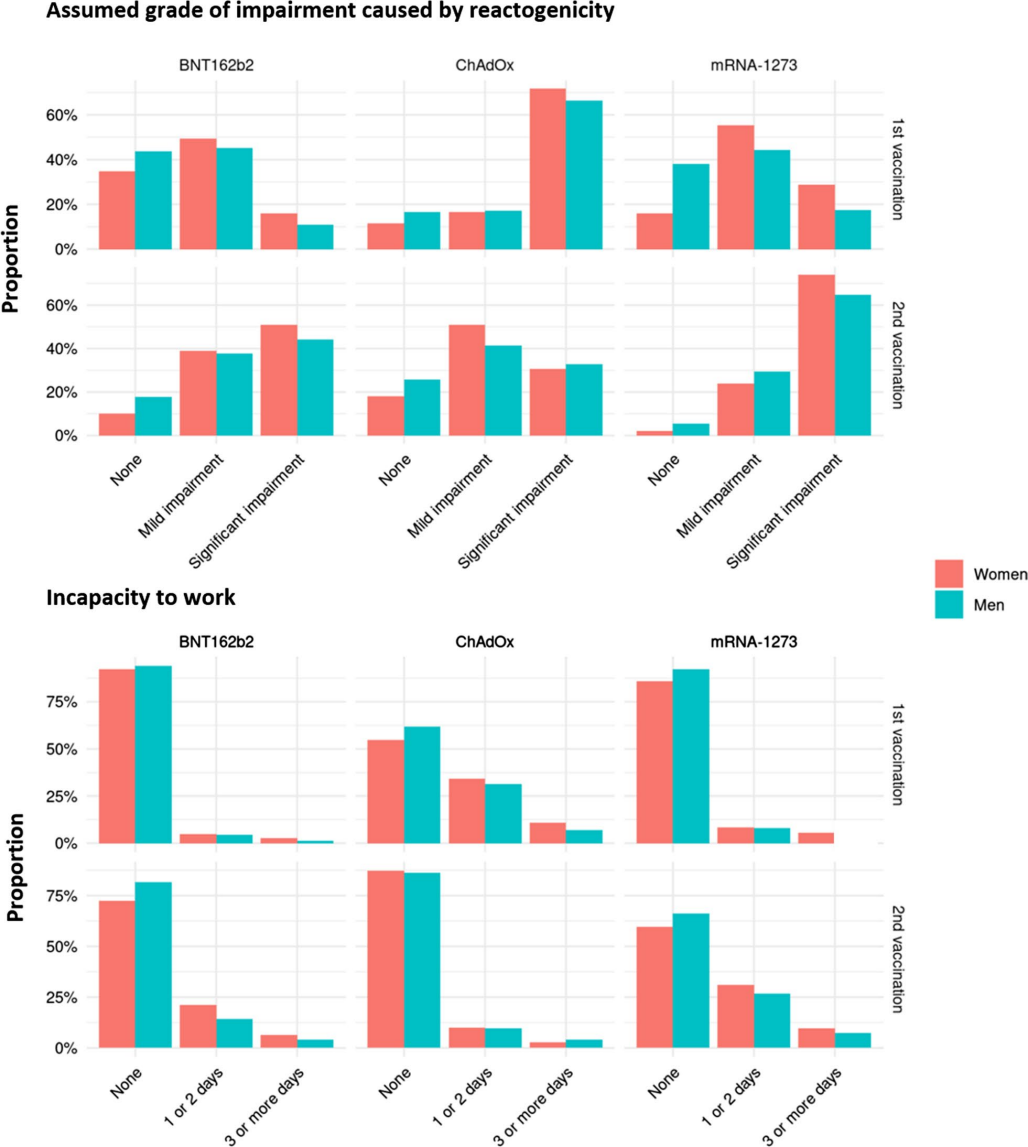
Reactogenicity and incapacity to work according to gender, vaccine and first versus second vaccination

The occurrence of both reactogenicity and of incapacity to work differed between genders, vaccines and between first and second vaccination (Fig. 2). According to the classification of the reported physical complaints, we assumed the highest rate of significant impairment after the first vaccination with ChAdOx (70.5%, 1938 of 2749 vaccinations with ChAdOx) and after the second vaccination with mRNA-1273 (71.6%, 358/500). In line with this, an incapacity to work was most often reported to have followed the first vaccination with ChAdOx (33.6%, 924/2749, 1–2 days, 10%, 275/2749, 3 or more days) and the second vaccination with mRNA-1273 (29.6%, 148/500, 1–2 days, 9.2%, 46/500, 3 or more days).

**Fig. 3:**
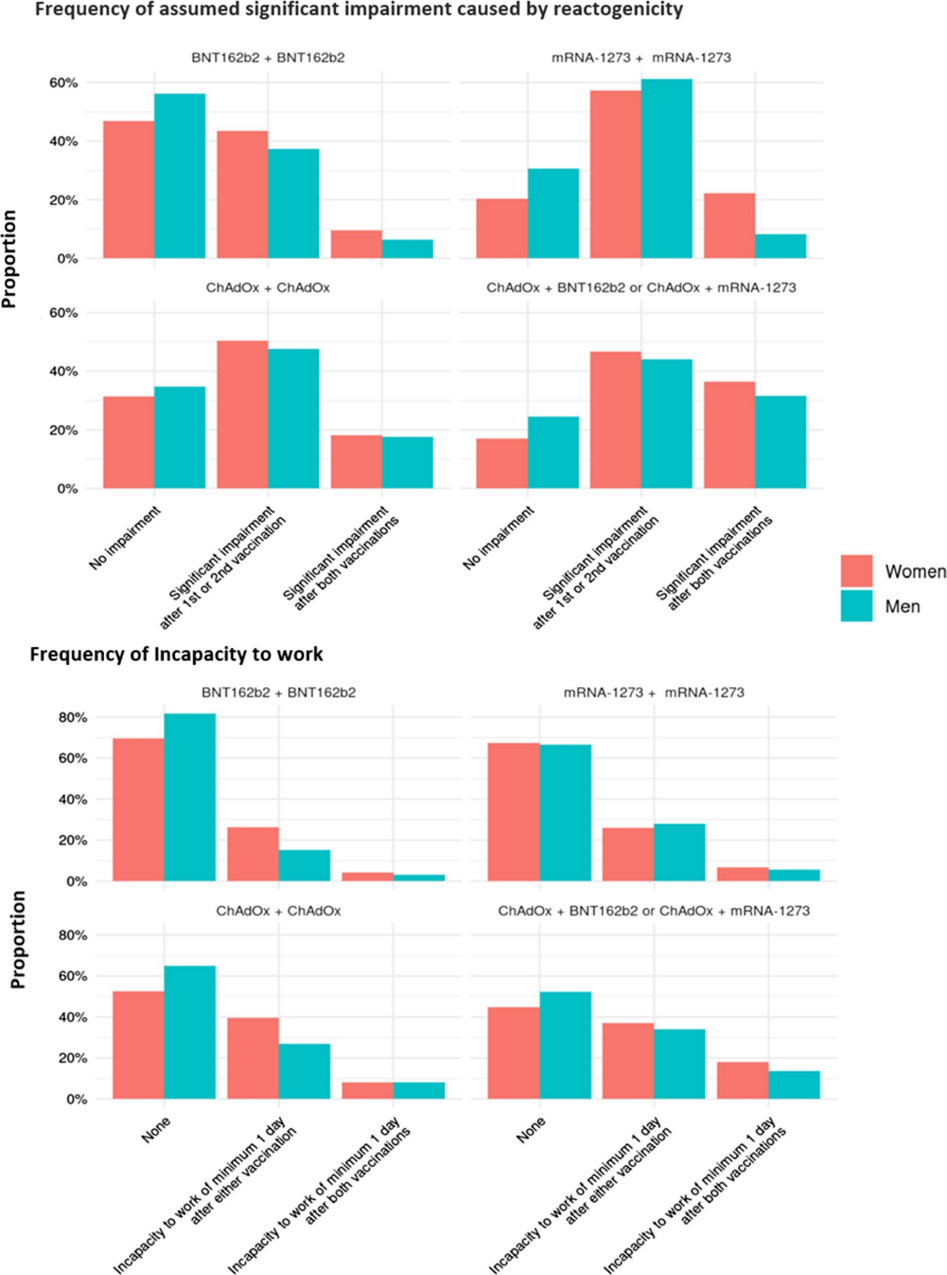
Reactogenicity and incapacity to work according to gender and vaccine combination

Figure 3 depicts the corresponding analysis focusing on vaccine combination as opposed to single vaccines. The homologue schedules with BNT162b2 showed the highest rate of participants reporting no major reactogenicity (49.4%, 2062 of 4170 homologue schedules with BNT162b2) or no incapacity to work (79.2%, 3302/4170) after either one of the vaccinations. Heterologous prime-booster vaccinations (ChAdOx followed by either mRNA-1273 or BNT162b2) were associated in 81.4% (1417 of 1740 heterologous schedules) with impairment after either one of the vaccinations; in 35.3% (615/ 1740) with assumed severe impairment after both vaccinations. An incapacity to work after both vaccinations was also most often reported for the same heterologous schedule (53.7%, 934/ 1740).

We conducted multivariable analyses addressing single vaccinations (Table 2) and their combinations (Table 3). Older age was independently associated with lower odds ratio for both impairing reactogenicity (e.g. > 61y versus 18–30y odds ratio OR 0.35) and loss of working days (e.g. > 61y versus 18–30y OR 0.50). The gender male was also associated with a lower risk for both, impairment (OR = 0.66) and loss of working days (OR = 0.72). The vaccination with ChAdOx or mRNA-1273 compared to BNT162b led to a two-fold increased odds. In the comparison of vaccine schedules, the heterologous combination ChAdOx + BNT162b or mRNA-1273 showed the highest risks (OR in comparison with the homologous schedule with mRNA-1273 for significant impairment 1.72, for loss of working days 1.57). The homologue schedules with ChAdOx and mRNA-1273 had a comparable risk (OR 1.43, 95% confidence interval 0.92–2.23). A homologue prime-booster vaccination with ChAdOx had more than a two-fold higher odds than the homologous schedule with BNT162b (OR for impairment = 2.48, for loss of working days 1.64).

**Table 2:** Multivariable analyses of risk factors for an impairment caused by reactogenicity or incapacity to work (single vaccinations)

Variable	OR (95% confidence interval)	*P* -value
Assumed grade of impairment caused by reactogenicity OR represents the odds ratio for a higher grade of impairment (i.e., significant vs. mild/none, significant/mild vs. none). Based on 12,387 vaccinations with complete information		
Age (31–40 vs. 18–30)	0.80 (0.71–0.90)	< .001
Age (41–50 vs. 18–30)	0.64 (0.57–0.71)	< .001
Age (51–60 vs. 18–30)	0.46 (0.41–0.52)	< .001
Age (> 61 vs. 18–30)	0.35 (0.30–0.41)	< .001
Time of vaccination (afternoon vs. morning)	1.04 (0.96–1.12)	ns
Male gender	0.66 (0.58–0.76)	< .001
Vaccination (second vs. first)	2.00 (1.75–2.29)	< .001
Vaccine (ChAdOx vs. BNT162b2)	2.43 (2.16–2.74)	< .001
Vaccine (mRNA-1273 vs. BNT162b2)	2.08 (1.72–2.51)	< .001
Male gender × Vaccination (second vs. first)	1.06 (0.89–1.25)	ns
Male gender × Vaccine (ChAdOx vs. BNT162b2)	1.03 (0.84–1.26)	ns
Male gender × Vaccine (mRNA-1273 vs. BNT162b2)	0.73 (0.50–1.06)	ns
Vaccination (second vs. first) × Vaccine (ChAdOx vs. BNT162b2)	0.05 (0.04–0.06)	< .001
Vaccination (second vs. first) × Vaccine (mRNA-1273 vs. BNT162b2)	1.50 (1.08–2.08)	.017
Duration of an incapacity to work OR represents the odds ratio for a longer incapacity to work (i.e., ≥ 3 days vs. 0–2 days, ≥ 1 days vs. none) Based on 11,105 vaccinations with complete information		
Age (31–40 vs. 18–30)	0.68 (0.58–0.78)	< .001
Age (41–50 vs. 18–30)	0.59 (0.51–0.67)	< .001
Age (51–60 vs. 18–30)	0.47 (0.41–0.54)	< .001
Age (> 61 vs. 18–30)	0.50 (0.40–0.62)	< .001
Time of vaccination (afternoon vs. morning)	1.08 (0.97–1.19)	ns
Male gender	0.72 (0.61–0.85)	< .001
Vaccination (second vs. first)	1.59 (1.30–1.96)	< .001
Vaccine (ChAdOx vs. BNT162b2)	2.12 (1.76–2.54)	< .001
Vaccine (mRNA-1273 vs. BNT162b2)	1.87 (1.42–2.46)	< .001
Male gender × vaccination (second vs. first)	0.88 (0.65–1.19)	ns
Male gender × vaccine (ChAdOx vs. BNT162b2)	1.33 (0.90–1.97)	ns
Male gender × vaccine (mRNA-1273 vs. BNT162b2)	1.17 (0.73–1.987)	ns
Vaccination (second vs. first) × vaccine (ChAdOx vs. BNT162b2)	0.05 (0.03–0.06)	< .001
Vaccination (second vs. first) × vaccine (mRNA-1273 vs. BNT162b2)	1.09 (0.68–1.75)	ns

**Table 3:** Multivariable analyses of risk factors for an impairment caused by reactogenicity or incapacity to work (prime-booster vaccinations)

Variable	OR (95% confidence interval)	*P* -value
Frequency of an assumed significant impairment caused by reactogenicity OR represents the odds ratio for a significant impairment after more vaccinations (i.e., after both vaccinations vs. after either one or none, after either one or both vaccinations vs. none) Based on 6747 vaccine combinations with complete information		
Age (31–40 vs. 18–30)	0.77 (0.66–0.90)	.001
Age (41–50 vs. 18–30)	0.64 (0.55–0.75)	< .001
Age (51–60 vs. 18–30)	0.47 (0.40–0.55)	< .001
Age (> 61 vs. 18–30)	0.34 (0.27–0.42)	< .001
Male gender	0.73 (0.61–0.87)	< .001
Vaccine combination (ChAdOx + ChAdOx vs. BNT162b2 + BNT162b2)	2.48 (2.08–2.94)	< .001
Frequency of an assumed significant impairment caused by reactogenicity OR represents the odds ratio for a significant impairment after more vaccinations (i.e., after both vaccinations vs. after either one or none, after either one or both vaccinations vs. none) Based on 6747 vaccine combinations with complete information		
Age (31–40 vs. 18–30)	0.77 (0.66–0.90)	.001
Age (41–50 vs. 18–30)	0.64 (0.55–0.75)	< .001
Age (51–60 vs. 18–30)	0.47 (0.40–0.55)	< .001
Age (> 61 vs. 18–30)	0.34 (0.27–0.42)	< .001
Male gender	0.73 (0.61–0.87)	< .001
Vaccine combination (ChAdOx + ChAdOx vs. BNT162b2 + BNT162b2)	2.48 (2.08–2.94)	< .001
Vaccine combination (mRNA-1273 + mRNA-1273 vs. ChAdOx + ChAdOx)	1.13 (0.08–1.58)	ns
Vaccine combination (ChAdOx + BNT162b/ mRNA-1273 vs. mRNA-1273 + mRNA-1273)	1.72 (1.25–2.37)	< .001
Male gender × vaccine combination (ChAdOx + ChAdOx vs. BNT162b2 + BNT162b2)	1.28 (0.91–1.80)	ns
Male gender × vaccine combination (mRNA-1273 + mRNA-1273 vs. ChAdOx + ChAdOx)	0.75 (0.38–1.49)	ns
Male gender × vaccine combination (ChAdOx + BNT162b/ mRNA-1273 vs. mRNA-1273 + mRNA-1273)	1.06 (0.56–2.01)	ns
Frequency of incapacity to work of one or more days OR represents the odds ratio for incapacity to after more vaccinations (i.e., after both vaccinations vs. after either one or none, after either one or both vaccinations vs. none) on 5153 vaccine combinations with complete information		
Age (31–40 vs. 18–30)	0.72 (0.60–0.86)	< .001
Age (41–50 vs. 18–30)	0.56 (0.47–0.67)	< .001
Age (51–60 vs. 18–30)	0.43 (0.36–0.51)	< .001
Age (> 61 vs. 18–30)	0,42 (0.31–0.56)	< .001
Male gender	0.73 (0.58–0.92)	.008
Vaccine combination (mRNA-1273 + mRNA-1273 vs. BNT162b2 + BNT162b2)	1.64 (1.09–2.46)	< .001
Vaccine combination (ChAdOx + ChAdOx vs. mRNA-1273 + mRNA-1273)	1.43 ( 0.92–2.23)	ns
Vaccine combination (ChAdOx + BNT162b/ mRNA-1273 vs. ChAdOx + ChAdOx)	1.57 (1.24–1.99)	< .001
Male gender × vaccine combination (mRNA-1273 + mRNA-1273 vs BNT162b2 + BNT162b2)	2.25 (1.00–5.06)	.050
Male gender × vaccine combination (ChAdOx + ChAdOx vs. mRNA-1273 + mRNA-1273)	0.51 (0.21–1.23)	ns
Male gender × vaccine combination (ChAdOx + BNT162b/ mRNA-1273 vs. ChAdOx + ChAdOx)	1.21 (0.76–1.94)	ns
Vaccine combination (mRNA-1273 + mRNA-1273 vs. ChAdOx + ChAdOx)	1.13 (0.08–1.58)	ns
Vaccine combination (ChAdOx + BNT162b/ mRNA-1273 vs. mRNA-1273 + mRNA-1273)	1.72 (1.25–2.37)	< .001
Male gender × vaccine combination (ChAdOx + ChAdOx vs. BNT162b2 + BNT162b2)	1.28 (0.91–1.80)	ns
Male gender × vaccine combination (mRNA-1273 + mRNA-1273 vs. ChAdOx + ChAdOx)	0.75 (0.38–1.49)	ns
Male gender × vaccine combination (ChAdOx + BNT162b/ mRNA-1273 vs. mRNA-1273 + mRNA-1273)	1.06 (0.56–2.01)	ns
Frequency of incapacity to work of one or more days OR represents the odds ratio for incapacity to after more vaccinations (i.e., after both vaccinations vs. after either one or none, after either one or both vaccinations vs. none) on 5153 vaccine combinations with complete information		
Age (31–40 vs. 18–30)	0.72 (0.60–0.86)	< .001
Age (41–50 vs. 18–30)	0.56 (0.47–0.67)	< .001
Age (51–60 vs. 18–30)	0.43 (0.36–0.51)	< .001
Age (> 61 vs. 18–30)	0,42 (0.31–0.56)	< .001
Male gender	0.73 (0.58–0.92)	.008
Vaccine combination (mRNA-1273 + mRNA-1273 vs. BNT162b2 + BNT162b2)	1.64 (1.09–2.46)	< .001
Vaccine combination (ChAdOx + ChAdOx vs. mRNA-1273 + mRNA-1273)	1.43 (0.92–2.23)	ns
Vaccine combination (ChAdOx + BNT162b/ mRNA-1273 vs. ChAdOx + ChAdOx)	1.57 (1.24–1.99)	< .001
Male gender × vaccine combination (mRNA-1273 + mRNA-1273 vs BNT162b2 + BNT162b2)	2.25 (1.00–5.06)	.050
Male gender × vaccine combination (ChAdOx + ChAdOx vs. mRNA-1273 + mRNA-1273)	0.51 (0.21–1.23)	ns
Male gender × vaccine combination (ChAdOx + BNT162b/ mRNA-1273 vs. ChAdOx + ChAdOx)	1.21 (0.76–1.94)	ns

## Discussion

In this cohort, around 75% of the COVID-19 vaccinations led to reactogenicity and nearly 25% of the vaccinations led to one or more days of work loss. The risk factors identified were female gender, younger age and the administration of a vaccine other than BNT162b2. ChAdOx induced reactogenicity mainly after the prime vaccination, while mRNA-1273 led to more pronounced reactions after boostering. Heterologous prime-booster vaccinations with ChAdOx followed by either mRNA-1273 or BNT162b2 were associated with the highest risk for impairments. The time of day of the vaccinations showed no influence.

The effect of age on reactogenicity has already been described in previous large-scale studies [5, 6, 15]. Data on sex or gender differences are still scarce, as results of previous trials were not reported in a gender-sensitive manner. In an analysis of the Centers for Disease Control and Prevention based on 13.7 million COVID-19 vaccine doses given in the USA, 79% of all adverse effects reported to the agency came from women, even though only 61% of the vaccines had been administered to them [9]. Further studies on real-world data showed a similar tendency towards a higher reactogenicity in females [8, 11].

Sex-related differences in the occurrence of adverse reactions have been disclosed for several drugs, with females suffering more often from side effects than males [16]. Until the early 1990s, women of childbearing age were kept out of drug trials in order to avoid exposing unknown pregnant women to drugs. Regimens developed on men were applied on women without further research on the possible effects of sex on a drug’s efficacy and safety. In consequence, 80% of drug withdrawals from the U.S. market were ascribable to new health risks found in women [17]. This gender discrepancy in side effects cannot only be explained by differences in pharmacokinetics [18], but also in pharmacodynamics, as shown by the higher risk for QT-prolongation [19].

Sex and gender differences influence immune response and outcome to infectious diseases. In general, females tend to have an enhanced immune response compared to males, which is held responsible for the higher propensity for developing autoimmune disease [20]. They have a more intense cellular and humoral immune response to vaccinations [21, 22], resulting in both, higher efficacy and more adverse effects [23] including anaphylactic reactions (24). One underlying mechanism is the sex hormone modulation of the immune system: antibody responses after influenza vaccine have been shown to be positively associated with concentrations of estradiol [25]. However, sex differential effects of vaccines remain distinct in age [26], suggesting that genetic or other factors are likely to be involved.

The gender bias in our study is in line with earlier findings and was expected. It is all the more surprising, that the vaccine approval studies included a sufficient proportion of females, but did not disaggregate safety and efficacy according to sex or gender. As published recently, only 17.8% of the COVID-19 related clinical trials published in scientific journals until December 15, 2020 reported sex-disaggregated results or subgroup analyses [27].

In our cohort, the distribution of the different vaccines was not homogenous between men and women: men received BNT162b2 more often, while women were vaccinated more often with ChAdOx. The reason can only be speculated: the prioritization of specific professional groups in the beginning of the vaccination program, the gender distribution in these professional groups and the availability of the different vaccines over time might have played a role. Given that both, female gender and ChAdOx are associated with more reactogenicity, this will have led to an increased total occurrence of reactogenicity and incapacity to work.

Nearly half of the participants were unable to work at least one day after the first vaccination with ChAdOx; a comparable rate was reported for the booster vaccination with mRNA-1273. A similar survey among health care workers found even higher rates of 65.3% after ChAdOx prime and of 56.8% after mRNA-1273 booster [8]. These rates are substantially higher than what could be expected according to the approval studies. The lack of personnel on such a large scale can endanger patient care and needs to be planned for when simultaneously vaccinating complete departments.

Why a vaccination with BNT162b2 in our study led to less reactogenicity and incapacity to work than the other two vaccines, is unclear. By the time of the survey, the image of ChAdOx had suffered from reports of major complications; this might have led to negative expectations and a higher awareness of vaccine reactions. However, this was not the case for mRNA-1273. A higher reactogenicity after mRNA-1273 compared to BNT162b2 has been described before; possible mechanisms were seen in higher dosage of mRNA-1273 (100 µg versus 30 µg mRNA) or in different RNA modifications [8]. Our study confirmed previous findings that while ChAdOx causes more reactogenicity at the prime immunisation, mRNA vaccines have more reactions effects at the booster [5, 6, 15]. Previous studies on safety of heterologous prime-boost COVID-19 vaccinations have shown diverging results. A participant-masked, randomised trial, found that heterologous vaccine schedules induced greater systemic reactogenicity following the boost dose than their homologous counterparts [10]. An analysis of a survey among 1313 vaccinated persons showed that individuals who received heterologous prime-boost schedules were more likely to report severe reactogenicity after their second dose [11]. Other studies from Germany have reported no difference in reactogenicity between homologous and heterologous schedules [12, 13].

Interestingly, we were not able to detect any influence of the time of day of vaccination. Interactions between vaccination timing and immunity response have been described before [28, 29, 30]; therefore, we were expecting an effect on the reactogenicity.

### Strength and limitations of this study

The strength of our study is the use of real-world data to assess reactogenicity and incapacity to work after different COVID-19 vaccines and schedules. The gender differences shown add evidence to an under-explored field; further studies addressing sex and gender specific immune responses to vaccines are needed.

The study suffers from several limitations. At the time of the survey, due to data protection rules, employers were not allowed to collect information on vaccinations among their employees. Therefore, there was no information on vaccination rate among staff. The survey addressed all vaccinated employees of the Helios group without knowing their exact number: the response rate among vaccinated employees could not be determined. The response rate of a survey presents a measure of the representativeness of the results, which however cannot be assessed here.

The cohort resulted from a convenience sampling, which limits the representativeness of the results. The addressed population—working-age persons with a high proportion of women—might have caused a bias. The decision as to whether or not to participate to this survey might have depended on several reasons; persons who suffered from stronger vaccine reactions might have been more prone to share their experiences, leading to an overestimation of reactogenicity. We did not record the time interval between prime immunisation, booster and survey. The recommended interval varied between vaccines; the vaccine program was an ongoing process. Time between vaccinations and survey might have varied substantially between participants, potentially influencing the memories pf reactions.

This study targeted common reactogenicity; it was not powered to assess rare and serious adverse effects.

We assessed gender according the information given in the questionnaire, which reflects the personal identification rather than the biological/genetic status. Therefore, the results of our study refer to gender, women and men rather than to sex, females and males, which needs to be observed when comparing with other studies.

## Conclusions

Young women reported the highest rates of reactogenicity after a COVID-19 vaccination, especially when vaccinated with a vaccine other than BNT162b2. When vaccinating a large part of a workforce, especially in professions that have high proportion of females and those of younger age such as health care, employers have to expect a noticeable amount of absenteeism. Assuming vaccine effectiveness to be equivalent across the vaccine combinations, employees may prefer to receive a homologous prime-booster immunisation with BNT162b2 to minimize reactogenicity and reduce lost work days. The designs of vaccines and vaccine strategies need to be sex-specific. Furthermore, it should be mandatory to report and publish sex-related variables in vaccine approval trials.

## Data Availability

Helios Health and Helios Hospitals have strict rules regarding data sharing because of the fact that health data are a sensible data source and have ethical restrictions imposed due to concerns regarding privacy. That is why we do not want to share our data. Access to anonymized data that support the findings of this study are available on request from the Leipzig Heart Institute (www.leipzig-heart.de). Please direct queries to the data protection officer (Email: info@leipzig-heart.de) and refer to study "eCaRe-COVID19" (HCRI ID 2020–0369).
